# Using refractive optics to broaden the focus of an X-ray mirror

**DOI:** 10.1107/S1600577517006038

**Published:** 2017-05-30

**Authors:** David Laundy, Kawal Sawhney, Vishal Dhamgaye

**Affiliations:** a Diamond Light Source, Harwell Science and Innovation Campus, Didcot, Oxon OX11 0DE, UK; b Indus Synchrotrons Utilisation Division, Raja Ramanna Centre for Advanced Technology, Indore 452012, India

**Keywords:** X-ray, focusing, refractive optics

## Abstract

X-ray mirrors are widely used at synchrotron radiation sources for focusing X-rays into focal spots of size less than 1 µm. The ability of the beamline optics to change the size of this spot over a range up to tens of micrometres can be an advantage for many experiments such as X-ray microprobe and X-ray diffraction from micrometre-scale crystals. It is a requirement that the beam size change should be reproducible and it is often essential that the change should be rapid, for example taking less than 1 s, in order to allow high data collection rates at modern X-ray sources. In order to provide a controlled broadening of the focused spot of an X-ray mirror, a series of refractive optical elements have been fabricated and installed immediately before the mirror. By translation, a new refractive element is moved into the X-ray beam allowing a variation in the size of the focal spot in the focusing direction. Measurements using a set of prefabricated refractive structures with a test mirror showed that the focused beam size could be varied from less than 1 µm to over 10 µm for X-rays in the energy range 10–20 keV. As the optics is in-line with the X-ray beam, there is no effect on the centroid position of the focus. Accurate positioning of the refractive optics ensures reproducibility in the focused beam profile and no additional re-alignment of the optics is required.

## Abstract 2

Refractive optical elements for X-rays that vary the size of the focal spot of an X-ray mirror are reported.

## 1. Introduction   

X-ray optical elements such as X-ray mirrors or refractive lenses are commonly used on synchrotron radiation beamlines to focus X-ray beams down to the sub-micrometre level for high-spatial-resolution microprobe experiments and for X-ray diffraction measurements from small crystalline samples. It is frequently desirable to be able to broaden the focus size in order, for example, to allow larger sample areas to be imaged more rapidly at lower spatial resolution or to allow the beam size to be matched to a diffracting crystal in order to reduce the sample radiation dose and minimize radiation damage during a measurement. Ideally this should be done with no significant reduction in the overall beam intensity in the focal spot and the change should be fast in order to meet the demands of rapid data collection at modern synchrotron radiation sources.

Existing methods for increasing the focus size include changing the surface profile of a bimorph mirror in order to change the mirror focal length (Alianelli *et al.*, 2016 ▸), using a combination of variable focus mirrors or compound refractive lenses to achieve a zoom configuration (Matsuyama *et al.*, 2016 ▸; Evans *et al.*, 2007 ▸; Schneider *et al.*, 2013 ▸) or using slits to change the size of the secondary source (Fischetti *et al.*, 2013 ▸). The first method is simple to implement but is liable to producing caustic-like structures on the focal spot (Sutter *et al.*, 2014 ▸). The second method presents a significant complication to the beamline design requiring an extra focusing optical element for each focusing direction with variable focal length in order to vary the magnification ratio. The third method requires additional optics to focus to the secondary source and there is a loss of X-ray intensity at the secondary slits. The time taken to change the focus size is limited in the first case by the time required for the bimorph mirror to stabilize after a change in the electrostatic potential of the piezo bending elements, and in the second case by the time for movement and realignment of the optics, and therefore beam size changes in less than 1 s are challenging to achieve.

In previous publications (Laundy *et al.*, 2015 ▸, 2016 ▸) we demonstrated a new concept in which the surface height profile of an elliptical mirror is deliberately modified in order to broaden the focused beam intensity profile in the direction of the mirror focusing. By applying the modification in separate parallel lanes running along the length of the mirror, we were able to demonstrate that the beam size could be reproducibly changed by translating the mirror laterally. This concept has recently been used to specify a seven-lane mirror for the VMXm macromolecular crystallography beamline at the Diamond Light Source, UK (Trincao *et al.*, 2015 ▸). This mirror will allow the beam size to be changed between seven predetermined values in the range 0.3–10 µm in a timescale of under 1 s. This capability will allow the diffraction from weakly diffracting samples to be rapidly measured in an automatic fashion while minimizing sample damage caused by exposure to the X-ray beam.

In a second publication we presented the use of hybrid optics (refractive plus reflective) in order to reduce the focus size of the optics of an X-ray beamline (Sawhney *et al.*, 2016 ▸). A refractive optical element is used to correct the X-ray wavefront for the distortion introduced by figure error on a focusing mirror.

In this paper, we extend this concept by employing refractive optics to broaden the focused beam profile of an elliptical X-ray mirror in a similar way that the profiled mirror works. We have manufactured refractive optical elements using the LIGA process, also known as deep X-ray lithography (Becker *et al.*, 1986 ▸), that can be installed shortly before the focusing mirror. The thickness profile of the refractive elements determines the X-ray path length modification that occurs on passing through the optical element and hence causes the perturbation of the wavefront that is required to achieve the required beam size. The principle is the same as that employed by X-ray compound refractive lenses to focus an X-ray beam; however, it is possible to design the refractive optics to expand the focused beam size to tens of micrometres with no aperture limit due to X-ray absorption. The LIGA process allows large numbers of devices made from the polymer SU8 to be made on a single silicon wafer substrate.

## 2. Theory   

The refractive index of matter in the X-ray regime at energy *E* is commonly written
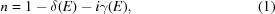
where 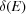 is the small energy-dependent deviation of the refractive index from unity which is responsible for a phase shift of the wavefront after passing through an optic, and 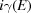 is the energy-dependent imaginary term responsible for absorption, *i.e.* the decay of the X-ray amplitude on passing through the optic. The wavefront path-length change caused by an X-ray passing at position *y* through an optic of thickness 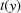 is
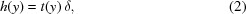
and the wave amplitude is also reduced by the factor 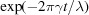. At X-ray energies, 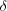 is of the order of 10^−5^ or less and, consequently, refraction is much weaker for X-rays than for visible light.

The distortion of the wavefront at the focusing optical element as a function of lateral position *y* can be written as 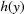, where 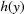 = 0 signifies zero distortion which gives the smallest, diffraction-limited, focal spot. The aim is to insert, into the X-ray beam path, a refractive structure that perturbs the wavefront, introducing a distortion 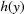 which, when propagated to the focal plane of the focusing optical element, results in a focus profile that is broadened by a known amount.

**Figure 1:**
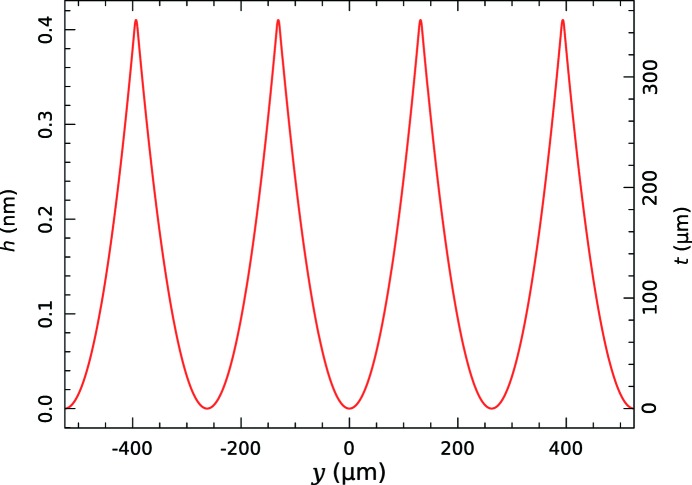
Wavefront perturbation (*h*) and SU8 refractive structure thickness (*t*) *versus* transverse position at the plane of the focusing optical element to produce a broadening of the focus to 5 µm at an X-ray energy of 15 keV.

Within the limits of geometrical optics, the wavefront distortion 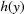 is equivalent to a slope error on the wavefront of 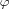 = 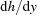 which causes a displacement at the focal plane, at a distance *Q* along the propagation direction, of *v* = 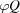. Two points on the initial wavefront separated by distance 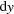 map, under geometric optics, onto two points at the focus separated by 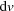 = 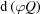 = 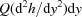, hence the requirement for uniform intensity distribution at the focus (*i.e.* a top-hat-like focus profile) can be met in the geometrical optics regime by 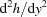 being constant so that uniform intensity on the initial wavefront gives uniform intensity at the focus. By direct integration, this is equivalent to the condition 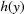 = 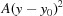 with *A* and 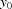 being constants. This can be achieved by splitting the wavefront at the focusing element (mirror) into sections of width 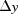, from 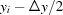 to 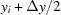 (*i* indexes the wavefront section) and ensuring that within the *i*th section the wavefront distortion is given by
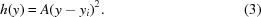
Then, for the wavefront from the *i*th section, the angle of the wavefront normal is 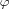 = 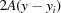. To obtain a target beam size *w* requires 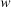 = 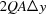 and therefore the parabolic coefficient of the wavefront is given by
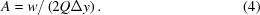
A calculated wavefront perturbation required to achieve a focused beam size of 5 µm at an X-ray energy of 15 keV and *Q* = 0.4 m is shown in Fig. 1 ▸. The wavefront has been split into five sections with parabolic arcs describing the required distortion in each section. A major advantage of splitting the wavefront into short sections is that the maximum required wavefront distortion varies as the square of the section width (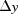) and this allows thinner refractive structures with lower X-ray absorption to be used.

While arguments based on geometrical optics can predict the size of the focal profile, physical optics which takes into account the wave nature of the X-rays is required to correctly model the intensity distribution for the focused highly coherent X-rays from a modern storage ring source. In each section of the initial wavefront 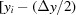 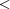 *y* 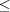 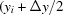)], using geometrical optics, each point (*y*) maps onto a distinct point at the focal plane and therefore each point on the focal plane receives a geometrical ray from a single point in each section of the initial wavefront. Physical optics, however, predicts that interference fringe structures caused by interference of the X-ray amplitude propagating from these spatially separated points will be present on the focal spot.

**Figure 2:**
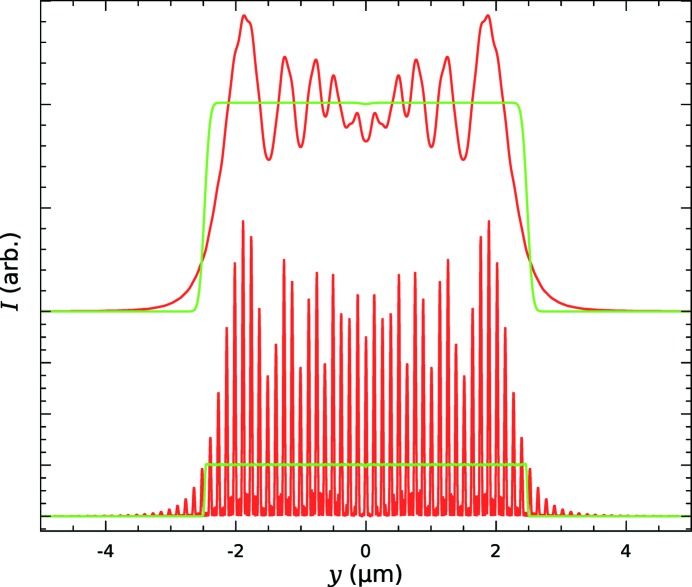
Simulated focus profile for a one-dimensional lens. A wavefront modified to give a focus beam size of 5 µm (green: geometrical optics; red: physical optics). Bottom: a coherent source. Top: an incoherent Gaussian profile source with r.m.s. size 8 µm (equivalent to an X-ray undulator source at the Diamond synchrotron). The X-ray energy is 15 keV, *Q* is 400 mm and *P* is 45 m.

It can easily be shown that two points separated by a distance 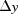 on the initial wavefront at the focusing optical element will produce fringes at the focus with periodicity of 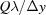 where 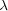 is the X-ray wavelength. The main features of the focus are a top-hat-like profile and the superimposed fringes caused by interference between different sections of the perturbed initial wavefront. This is verified by physical optics simulations using one-dimensional wavefront propagation based on the Fresnel–Kirchoff equation. Fig. 2 ▸ shows the simulated focus beam profile for a coherent source and a one-dimensional focusing lens calculated using geometrical optics and by wavefront propagation. The source size affects the profile due to incoherent summation across the demagnified source profile which results in convolution of the profile with a Gaussian profile of r.m.s. width 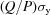 where 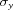 is the source r.m.s. size, *P* is the distance from source to the focusing optical element and *Q* is the distance from optic to the focus. The effect of the incoherent source is shown at the top of Fig. 2 ▸. The interference fringes with spacing 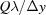 = 125 nm are clearly visible for the coherent source but they are smeared out by the incoherent source.

The width of each parabolic section of the initial wavefront at the focusing optical element (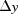) determines the fringe spacing on the focus profile (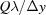), and if 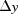 is too small then the fringe separation becomes large and the fringes are more apparent on the focus profile; however, if 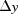 is large then the fringe spacing is smaller and is smeared out by the source convolution and other wavefront errors and instability. In addition, if 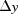 is too small, wavefront propagation simulations (Laundy *et al.*, 2015 ▸) showed that the wavefront modulation acts like a diffraction grating and intensity starts to appear in higher orders separated from the main focus.

In contrast, if 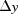 is large then the refractive optic required to produce the parabolic wavefront distortion must be made thicker and absorption may start to become significant especially at lower X-ray energies. Smaller 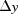, which splits the wavefront over a larger number of sections, will also have the advantage of reducing the effect of the mirror slope error in producing caustic structures at the focus because the mirror slope error variation within each wavefront section should be smaller and any caustic structures originating from different sections are unlikely to coincide.

For practical applications on synchrotron radiation beamlines, it is desirable that the refractive optics are positioned upstream of the focusing mirror. This is because space downstream of the mirror is usually at a premium, being taken by slits and apertures and also the sample environment. Upstream of the mirror, there is often space available for additional optics, and the longitudinal positioning of the refractive structures is not critical to their functioning. The wavefront perturbation generated by the refractive optics will be modified after reflection from the focusing mirror; however, if the mirror focal length is not too short compared with the mirror length, this effect will be small and the wavefront perturbation will not be significantly changed by reflection from the mirror.

In order to design the refractive structure to obtain a given focal spot size *w*, the amplitude of the parabolic sections (*A*) is calculated from equation (4) using 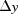, the wavefront section width. The wavefront distortion function 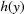 is then calculated using the amplitude *A* inserted into equation (3). This function is then converted to a thickness profile for the refractive object using the refractive index decrement (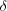) for the given X-ray energy *E* and equation (2). To add stability to the fabricated free-standing structures the thickness profile of each structure is increased by a constant amount (typically about 50−100 µm) and the profile of this structure is then added to a design layout drawing by positioning the structure in a free area.

For the test measurements, we required that the focused beam size should be varied in the range 1–10 µm at X-ray energies from 8 keV to 24 keV. This required a sequence of refractive optics with structures of varying amplitude, the smallest amplitude being required for achieving the smallest focused beam size at the lowest X-ray energy and the largest amplitude for achieving the largest focused beam size at the highest X-ray energy. The change in beam size could then be executed by translating the required refractive optical element into the X-ray beam.

## 3. Experiment   

The refractive optical elements made by the LIGA process are planar structures with near-vertical side walls and high aspect ratios. The structures are composed of the polymer SU8 which for X-rays has a favourable ratio of refraction to absorption and low surface roughness and they are well suited for X-ray refractive optics deflecting the X-rays within the plane of the structures. The structures are laid down on a flat single-crystal silicon wafer substrate allowing a range of structures to be inserted into the X-ray beam by translation of the wafer. The structures were initially drawn using CAD and then converted to a photo mask which was used to produce the LIGA structures. The fabrication process allows a large number of refractive optical elements (many hundreds) to be laid out on a wafer. In our initial test design, we arranged the structures in ten columns on the 100 mm wafer which were then separated by slicing the wafer into ten strips, each containing about 20 refractive optical elements arranged on a vertical line along the length of each silicon wafer strip. A wafer strip could be mounted in the X-ray beam with horizontal translation and vertical translation being used to position the selected refractive optic in the X-ray beam. The initial structures had a depth of about 200 µm; however, it is expected that this will be increased in the future.

**Figure 3:**
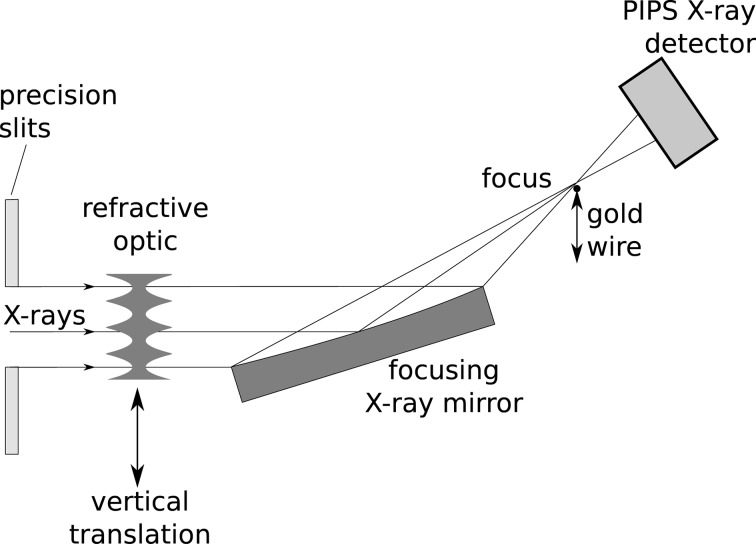
Schematic of the experimental arrangement.

Measurements were made using the Diamond Light Source Test Beamline (Sawhney *et al.*, 2010 ▸). On this beamline the X-rays from the dipole source are monochromated by a double-crystal silicon monochromator. The experimental arrangement of the mirror in the experimental hutch is shown schematically in Fig. 3 ▸. The monochromatic beam cross section was selected by a set of precision four-blade slits at about 45 m from the source. The focusing mirror was a 90 mm-long vertically deflecting elliptically profiled mirror with focal length of 400 mm, an incidence angle of 3 mrad and with a rhodium coating on the reflecting surface giving total external reflection of the X-rays to energies above 20 keV. At this low incidence angle the mirror collected and focused a beam aperture of 270 µm giving a horizontal line focus 400 mm downstream from the mirror centre and 2.4 mm above the incident beam direction. The refractive optics is located about 1 m upstream of the mirror and a vertical translation allows different refractive structures to be translated and positioned in the X-ray beam.

The beam size at the focal plane is measured by scanning a thin horizontally orientated gold wire vertically through the beam focus using a piezo translating stage while measuring the transmitted X-ray intensity using a passive implanted planar silicon (PIPS) integrating X-ray detector. The intensity distribution of the beam is then found by taking the intensity difference at each step of the wire. The mirror had been characterized previously by Diamond NOM (Alcock *et al.*, 2010 ▸) and also by using at-wavelength metrology. The focal spot size of the mirror was measured to be 0.5 µm (FWHM), the main contribution being the demagnified source size at the Test Beamline (source vertical r.m.s. size 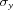 = 20 µm).

The mirror was mounted on a goniometer with fine pitch control and additional yaw and roll adjustment. Measurements were made for a range of focused beam sizes at three X-ray energies.

## 4. Results   

**Figure 4:**
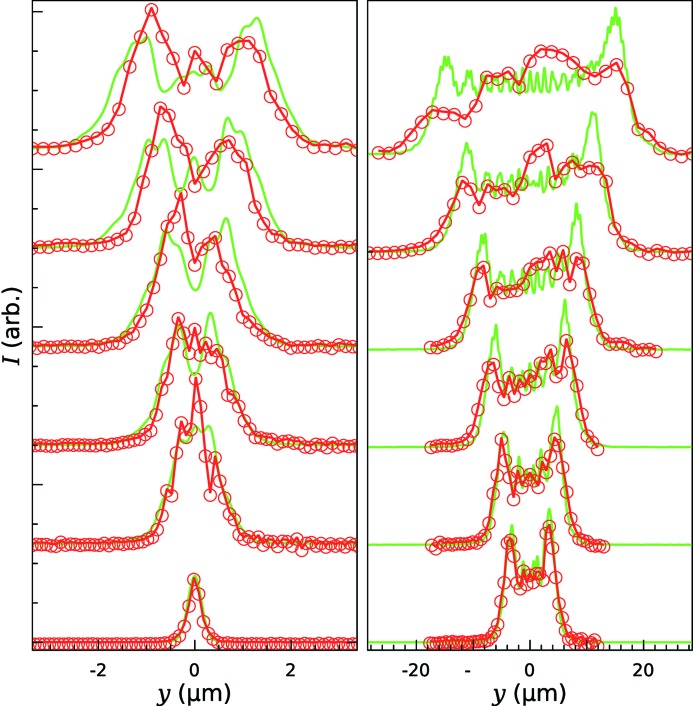
Measured focus profile (red) with the simulated profile (green). Left: broadening in the range up to 5 µm; right: broadening in the 5–20 µm range. The step size was varied from 100 nm for the narrowest (unbroadened) profile to 1 µm for the most broadened profile.

**Figure 5:**
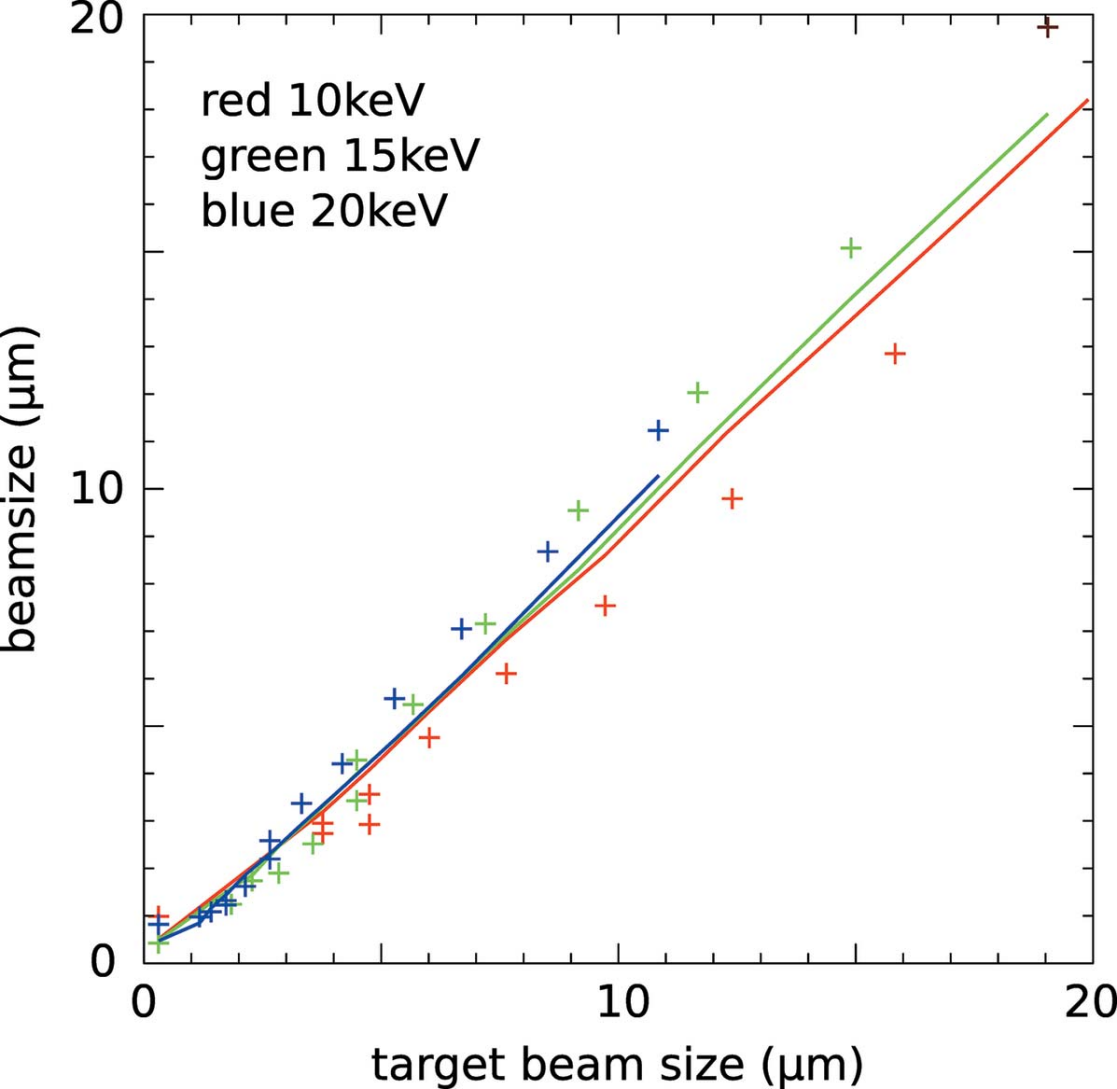
Relationship between the calculated beam size and the target beam size (*w*) at 10, 15 and 20 keV, from measurement (points) and from simulation (lines). The beam size was defined as the full width at one-fifth maximum.

Fig. 4 ▸ shows a series of measurements made at 15 keV of the profile of the focused X-ray beam with a series of refractors inserted into the X-ray beam. Also shown are simulated profiles made using one-dimensional wavefront propagation. Similar measurements were made at X-ray energies of 10 keV and 20 keV. The asymmetry of the measured and calculated intensity profiles is caused by the variation of the strength of the focusing along the length of the mirror which is at a low glancing angle to the beam. Fig. 5 ▸ shows the measured and simulated widths of the focus profiles as a function of the target focus size (*w*) used to calculate the profile of the refractive structure [equation (4)]. In addition, the integrated X-ray transmission through the structures was calculated and was found to be in excess of 95% even for the thickest structure at the lowest X-ray energy (10 keV).

## 5. Conclusions   

This new optics will find applications for experiments at X-ray sources where a variable focused beam size is an advantage. Macromolecular crystallography experiments require the ability to match the focused beam size to the size of the crystal sample in order to minimize radiation damage. For micro­probe experiments a variable-sized probe would allow different length scales to be measured efficiently. For diffraction experiments from polycrystalline samples an increased focused beam size could be used to improve sample averaging over crystallite orientations.

The results clearly show that these fabricated refractive structures, which deliberately modify the X-ray wavefront, can be used to provide a variable-sized focused beam. The features of this new optics are:

(i) Simplicity of implementation: the optics is in-line with the X-ray beam and requires only a small longitudinal space before the focusing mirror. The exact position along the beamline is not critical for the operation of the devices. This would allow the devices to be installed as an upgrade to an existing beamline.

(ii) The optical axis does not change when the devices are inserted. As a result, the focus position does not move as the refractive structures are moved into the X-ray beam and no realignment of the focusing optics is required.

(iii) Low cost. The fabrication process is well established and is well suited to making custom designs. Using wafer substrates of diameter 3 inches allows hundreds to thousands of unique structures to be fabricated. The alignment requires two translations and two rotation axes. A standard mounting for the devices would allow the structures to be easily replaced and upgraded as design and fabrication improves.

(iv) Having the possibility of a large number of available structures gives a quasi-continuous control of the beam size.

(v) The X-ray absorption in the structures is low. The highest absorption occurs at the lowest X-ray energy for the largest amplitude structures needed for achieving the highest broadening. Even at 10 keV and for a focused beam size of 20 µm the X-ray transmission was over 95%. Future designs would be able to reduce the amplitude of the structures by adopting a shorter repeat period.

(vi) Fast changes in beam size are possible. With suitable goniometry, it will be possible to move the structures into position in significantly less than 1 s. This will be essential in order to meet the demands for automated high-throughput macromolecular crystallography experiments when the beam size must be altered to match each crystal sample (Evans *et al.*, 2011 ▸).

While the focus broadening from a given structure is energy dependent because of the dispersion in the refractive index, we have shown that a sequence of structures of increasing amplitude can be used to generate a range of beam sizes from 1 µm to over 10 µm over an energy band of 10–20 keV on the Diamond Test Beamline. The same principle could be used with thinner LIGA structures to broaden the sub-100 nm focus size on a nano-probe beamline to cover the range to 1 µm and we expect future generations of the structures to also extend the broadening range to beyond 20 µm. It is also our aim to increase the depth of the structures to cover the aperture of a two-dimensional focusing optic such as a Kirkpatrick–Baez mirror system. This will allow the beam size to be independently controlled in the horizontal as well as vertical directions using two crossed refractive structures in-line with the X-ray beam. The structures present on the focused beam profiles are predicted by the physical optics simulations and it is hoped that by subtly changing the profiles of the refractive structures, for example changing the width of each section 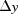 by different amounts, a more uniform focal profile may be obtained.
